# Renormalized charge and dielectric effects in colloidal interactions: a numerical solution of the nonlinear Poisson–Boltzmann equation for unknown boundary conditions

**DOI:** 10.1140/epje/s10189-023-00334-2

**Published:** 2023-09-11

**Authors:** Alexander Schlaich, Sandeep Tyagi, Stefan Kesselheim, Marcello Sega, Christian Holm

**Affiliations:** 1 Stuttgart Center for Simulation Science (SC SimTech), University of Stuttgart, 70569 Stuttgart, Germany; 2 Institute for Computational Physics, University of Stuttgart, Allmandring 3, 70569 Stuttgart, Germany; 3 2a Malzeard Road Surgery, Luton, LU3 1BD UK; 4 Forschungszentrum Jülich (FZJ), Jülich Supercomputing Centre, 52428 Jülich, Germany; 5 Department of Chemical Engineering, University College London, London, WC1E 7JE UK

## Abstract

**Abstract:**

The Derjaguin–Landau–Verwey–Overbeek (DLVO) theory, introduced more than 70 years ago, is a hallmark of colloidal particle modeling. For highly charged particles in the dilute regime, it is often supplemented by Alexander’s prescription (Alexander et al. in J Chem Phys 80:5776, 1984) for using a renormalized charge. Here, we solve the problem of the interaction between two charged colloids at finite ionic strength, including dielectric mismatch effects, using an efficient numerical scheme to solve the nonlinear Poisson–Boltzmann (NPB) equation with unknown boundary conditions. Our results perfectly match the analytical predictions for the renormalized charge by Trizac and coworkers (Aubouy et al. in J Phys A 36:5835, 2003). Moreover, they allow us to reinterpret previous molecular dynamics (MD) simulation results by Kreer et al. (Phys Rev E 74:021401, 2006), rendering them now in agreement with the expected behavior. We furthermore find that the influence of polarization becomes important only when the Debye layers overlap significantly.

**Graphical Abstract:**

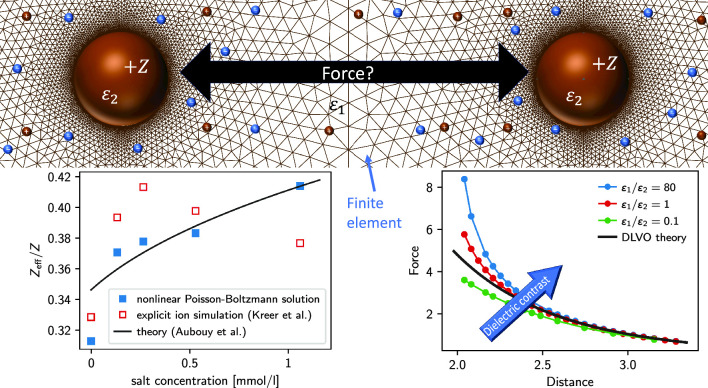

## Introduction

Colloidal dispersions in aqueous solutions have been the subject of numerous studies [1] because they play a role in industrial processes involving coatings, aerosols, or ceramics and for separation or filter processes [2]. They are also an excellent model system for fundamental soft-matter research as one can select their charge, and thus interaction range and strength, over a wide range of values [3]. One notable example is that of charge-stabilized colloidal suspensions, commonly used to study crystallization [4, 5]. Pairwise interactions, as predicted by the Derjaguin–Landau–Verwey–Overbeek (DLVO) theory [6, 7], proved successful in explaining experimental measurements using optical tweezers [8], magnetic chaining [9], atomic force microscopy [10, 11] or laser radiation pressure methods [12]. Within this framework, electrolytes in solution play a fundamental role by screening the electrostatic interactions between the colloids and governing the effective interactions. The case of monovalent mobile ions is significant from the theoretical perspective because it is amenable to a mean-field level description, where one combines the Poisson equation of electrostatics with the Boltzmann distribution of the mobile ions to obtain the nonlinear Poisson–Boltzmann (NPB) equation [13, 14]. The NPB equation can be used to determine the electrostatic potential for many problems, but its solution is typically computationally demanding. An analytical solution of the NPB equation exists only for the case of an electrolyte solution in contact with an infinite plane, as Gouy and Chapman showed more than a century ago [15, 16], and other problems require a numerical solution. Typical approaches include finite differences [17], finite element schemes [18], or multi-grid methods [19], and have recently been extended to machine learning methods [20]. A complication arises for cases with nontrivial boundary conditions. The solution of the NPB equation for the case of two interacting charged spheres, for example, has been the subject of many studies during the 1980s and 1990s, although usually employing constant potential or constant electric field boundary conditions [21–23].

In the case of particles with a low surface charge (when the electrostatic energy $e\psi$ is sufficiently small compared to the thermal energy $k_{\textrm{B}} T$), one can linearize the NPB equation, obtaining an effective interaction potential via the DLVO theory. In their famous work, Alexander and coworkers proposed using the DLVO potential even for highly charged particles at low colloid packing fraction, provided that a renormalized charge is used [24]. What is now known as Alexander’s prescription is a numerical recipe that requires solving the NPB equation and fitting a DLVO potential sufficiently far away from the particle, where linearization is appropriate. By integrating the resulting effective potential up to the colloid’s surface, one obtains an effective charge, typically smaller than the bare one. In 2003, Aubouy and coworkers [25] proposed an analytical estimate for the renormalized charge in the colloid infinite dilution limit that has shown to be of great value to the community and fully agrees with the numerical solutions of the NPB equation we present in this article. We here limit to the case of relatively low colloid packing fractions $\eta =4 \pi \rho _\textrm{p}a^3/3$, where $\rho _\textrm{p}$ is the colloid particle density in the electrolyte solution and *a* their respective radius. In that case, Alexander’s prescription and the analytical estimate by Aubouy et al.—which is derived for the zero volume fraction limit $\eta =0$—is expected to hold. In the more general case, a Wigner–Seitz cell model of the NPB equation has to be considered, i.e., the potential—or counter-ion excess concentration—will not decay to zero but remain at a finite value. The effective charge then has to be determined self-consistently [26–28]. In this renormalized Jellium model approach, where the effective charge is consistent with Alexander’s prescription, the DLVO potential is exact in the far field and the potential of mean force agrees with explicit ion Monte-Carlo simulations [29].

When the double layers start to overlap (i.e., when the particles come close together), the typically employed boundary condition assuming a constant surface charge on the colloid’s surface becomes questionable as the effective charge density on the surface will be affected by the electrostatic repulsion of the other particles. In this case, one must pay attention to polarization effects, and one cannot assume a homogeneous potential on the particle’s surface. Whereas several numerical methods to effectively model the interaction of two charged spheres exist, they typically rely either on the approach of constant surface charges [21] or constant surface potential [30] and often use linearized Poisson–Boltzmann theory or some other kind of simplification. Further approximations appear when the jump of the dielectric constant between the low dielectric particles and the high dielectric solvent is included into the mean-field approximation, such as linearized perturbation theory [31], or introducing a nonlinear dielectric response into the canonical partition function, resulting in a dipolar Poisson–Boltzmann equation [32]. Further complication would arise if one would include the effects of charge regulation for pH-dependent ion dissociation. For this field, new analytical results [33–35] and simulation algorithms [36–38] have appeared, revitalizing the interest in pH-sensitive interaction scenarios. For simplicity, we will not discuss this interesting additional complication in the present article.

Concerning the case of colloidal particles, the boundary condition at the particles’ surface is in general unknown. A possibility to determine it is to use continuity conditions and solve Poisson’s equation in the particles interior [18], but this usually leads to numerical problems since already tiny inaccuracies in the numerical solution of the Poisson problem can result in large errors for the total potential. Here, we use an iterative procedure to determine the correct boundary conditions and present the solution of the two-colloid problem with dielectric mismatch, showing that one can use the effective DLVO potential to model the system using the analytical estimates of Aubouy and coworkers. In light of our results, we reinterpret the explicit ion molecular dynamics (MD) results of Kreer et al. [39] on the effective charge of a pair of colloids, rendering their findings less surprising. Examining the impact of the dielectric contrast between solvent and colloidal particles, we show that the interaction energy drastically changes when the Debye layers strongly overlap, such as is the case for dense suspensions at low salt concentrations. These deviations greatly exceed the inaccuracy of DLVO approximation and can be explained qualitatively in terms of image charges contribution.

## Poisson–Boltzmann equation with unknown boundary conditions

When the correlations between micro-ions in a medium of homogeneous dielectric constant $\epsilon$ can be neglected, one can describe the system by combining the Poisson equation with the Boltzmann probability distribution, resulting in the so-called NPB equation for the reduced electrostatic potential $\Phi =e\psi /(k_\textrm{B}T)$. Here, $\psi$ is the electrostatic potential, *e* the unit charge, $k_\textrm{B}$ Boltzmann constant, and *T* the absolute temperature. The NPB equation takes, in the general case and assuming a symmetric monovalent salt, the form

1 $$\begin{aligned} \nabla ^{2}\Phi (\varvec{r}) =\frac{1}{\lambda ^{2}}\sinh \Phi (\varvec{r}) -4\pi \ell _\textrm{B} \sum _i^{N_c} Z_i\delta \left( \varvec{r}-\varvec{r}_i\right) . \end{aligned}$$

Besides the mobile charges, described at the mean-field level, Eq. (1) includes the presence of $N_c$ point-like charges of valence $Z_i$ to represent the colloidal charges. The quantities $\lambda =1/\sqrt{4\pi \ell _\textrm{B} c_0}$ and $\ell _\textrm{B}=e^2/(\epsilon k_\textrm{B}T)$ denote the Debye and Bjerrum lengths, respectively, where $c_0$ is the bulk concentration of the electrolyte species.

The main problem that one has to face to solve the NPB equation using finite elements in the presence of one or more rigid objects (assumed to be impermeable to the ions) is that due to the excluded volume, a discontinuity in the electric field appears at the surface of the objects. In this case, one cannot solve the NPB equation in a single domain. In principle, one should solve the NPB equation in the outer region and the Laplace equation in the inner region, matching the boundary conditions at the surface of the objects in a self-consistent way. In practice, the inner-domain solution is rarely necessary. Therefore, one can reduce the problem to finding the NPB equation’s solution with an appropriate boundary condition at the surface of the colloids.

In the following formulation, we use Neumann boundary conditions, specifying the normal component of the electric field at the surface, $-\nabla \Phi _S \cdot {{\textbf {n}}} = {{\textbf {E}}}_S\cdot {{\textbf {n}}} = j$, where *j* denotes the electric field flux through the surface. The key idea is to solve the problem of unknown boundary conditions by casting the NPB equation into an implicit form,

2 $$\begin{aligned} \varvec{E}_S(\varvec{r}) = - \nabla \Phi _S(\varvec{r}) = f \left( \rho ( \Phi ) \right) , \qquad \varvec{r} \in S. \end{aligned}$$

The functional *f* can take different forms, depending on the geometry and symmetries in the system that affect the corresponding Green’s function. Here, $\Phi$ and $\Phi _S$ denote the potential in the whole domain and on the boundary surface, respectively, and ${{\textbf {E}}}$ and ${{\textbf {E}}}_S$ are the corresponding electric fields. Finally, the electric charge density $\rho$ depends implicitly on the potential $\Phi$ within the mean-field approximation.

The implicit formulation can be written, in the most general case, as the solution for the Poisson equation associated with the NPB one:

3 $$\begin{aligned} \varvec{E}_S(\varvec{r})= & {} \, \ell _\textrm{B} \sum _i^{N_c} \frac{Z_ie \left( \varvec{r}-\varvec{r}_i\right) }{|\varvec{r}-\varvec{r}_i|^3}\nonumber \\{} & {} + \frac{1}{4\pi {}\lambda ^2}\int {{\sinh \Phi (\varvec{r}^\prime )}\frac{{{\textbf {r}}}-{{\textbf {r}}}^\prime }{|\varvec{r}-\varvec{r}^\prime |^3} \text {d}\varvec{r}^\prime }, \end{aligned}$$

where the term proportional to $\sinh (\Phi )$ is regarded as the (unknown) mean-field charge distribution, and the position vectors $\varvec{r}_i$ and $\varvec{r}^\prime$ can span the whole domain of the problem.

Equation (3) is the starting point for the iterative approach, which can be implemented using one of the many available methods [40] including, for example, Jacobi, Gauss–Seidel, or successive over-relaxation (SOR). In the SOR approach, which we use in the calculation presented here, the equation ${{\textbf {E}}}_S = f(\Phi )$ is approximated to a desired accuracy by generating the sequence of estimates

$${{\textbf {E}}}_S^{(n+1)} = \alpha f(\Phi ^{(n)})+(1-\alpha ) {{\textbf {E}}}_S^{(n)}, $$

starting from an arbitrary (usually random) initial guess of the solution. The relaxation parameter, $\alpha \in (0,2)$, governs the convergence speed. For the numerical implementation, we employed the highly efficient DUNE framework [41] and rely on DUNE-PDELab and the GMSH finite element mesh generator [42] for discretization. The technical aspects can be found in Ref.  [43]. The various parts of our iterative Poisson Boltzmann solver (IPBS) can be summarized as follows:

1. Assign the value for the flux at any boundary, where this is known from physical or symmetry reasons.
2. Assign an arbitrary value for the flux at the surface of the colloids, $j^{(n)}_S$.
3. Solve the NPB equation using this boundary condition, and obtain the estimate for the electrostatic potential in the domain of computation, $\Phi ^{(n)}$.
4. Compute a new estimate for the electric field on the boundaries, ${{\textbf {E}}}^{n+1}_S$ applying Eq. (3) to $\Phi ^{(n)}$.
5. Iterate steps 2 and 3 until a desired relative accuracy $\Delta =\max \left\{ 2 \frac{|\Phi ^{n+1}_S-\Phi ^{(n)}_S|}{|\Phi ^{n+1}_S+\Phi ^{(n)}_S| }\right\}$ is obtained.

**Fig. 1:**
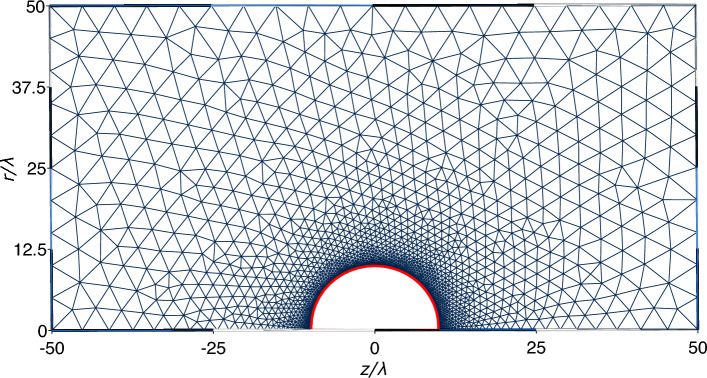
A typical mesh used in the finite element method employing the symmetry conditions for spherical particles. The mesh is highly resolved in the region close to the surface of the colloid (void space around the origin). The red line corresponds to the iteratively determined boundary condition

The IPBS scheme is easy to implement and of general applicability since it does not depend on which technique one uses to solve the NPB equation. One can reduce the problem of two colloids to a two-dimensional one by exploiting its symmetry. Working in cylindrical coordinates $(r,\phi ,z)$ and aligning the centers of the two colloids along the *z* axis, the solution does not depend anymore on the angle $\phi$, and the NPB equation reads then

4 $$\begin{aligned} \frac{\partial ^{2}\Phi }{\partial r^{2}}+\frac{\partial ^{2}\Phi }{\partial z^{2}}=\frac{1}{\lambda ^{2}}\sinh \Phi -\frac{1}{r}\frac{\partial \Phi }{\partial r}, \end{aligned}$$

where $\Phi =\Phi (r,z)$. Equation (4) is a nonlinear elliptic equation and can be solved employing standard finite elements methods [44] on a rectangular (*r*, *z*) domain ${\mathcal {D}}$. To do so, one needs to determine the unknown boundary conditions at the surface of the colloids and those on the border $\partial {\mathcal {D}}$ of the domain ${\mathcal {D}}$. Because of the electrostatic screening provided by the electrolyte, we know that the electric field should tend to zero far away from the colloid, and because of symmetry reasons, the normal component of the electric field has to be identically zero along the axis passing through the colloid centers.

By introducing a mesh of *N* nodes located at position $(r_j,z_j)$, the implicit solution of Eq. (4) can be written as

5 $$\begin{aligned} \Phi (r,z) =&\, \ell _\textrm{B} \sum _i^{N_c} \frac{Z_ie}{|\varvec{r}-\varvec{r}_i|} - \nonumber \\&-\frac{1}{4\pi \lambda ^{2}}\sum _{j=0} ^{N}\Theta _j \sinh \Phi (r_j,z_j), \end{aligned}$$

where

6 $$\begin{aligned} \Theta _j =\int _{0}^{2\pi }\frac{\text {d}\phi }{\sqrt{\left( z-z_{j}\right) ^2 + r^2 + r_j^2 + 2rr_j\cos \phi }}. \end{aligned}$$

In turn, one can express the integrals $\Theta _j$ in terms of the complete elliptic integral of the first kind [45] $K(m) = \int _0^{\pi /2}[ 1-m\sin ^2(q) ]dq$ as

7 $$\begin{aligned} \Theta _j = \frac{4}{\sqrt{\alpha _j-\beta _j}}K\left( \frac{2\beta _j}{\beta _j-\alpha _j}\right) , \end{aligned}$$

where $q=\phi /2$, $\alpha _j=(z-z_{j})^2 + r^2 + r_j^2$ and $\beta _j=2rr_j$. Equations (5) and (7) can eventually be used to compute the electric field, Eq. (3), on the boundary *S* and, therefore, to solve for the unknown boundary conditions using the IPBS algorithm.

## Algorithm validation and the two-colloid problem

Before applying IPBS to the two-colloid case, we tested it in the single colloid case, still employing the two-dimensional scheme described so far, to check its implementation. Although no analytical solution in closed form exists even for the simple problem of a single colloid (see Ref. [46] for a solution in the form of a series), one can still perform a quite stringent numerical check by comparing to the solution obtained with the Neumann boundary condition. Indeed, given the symmetry of the problem, the electric field has to be uniform on the colloid surface and can, therefore, be estimated using Gauss law. We have solved the problem of a colloid of charge $Ze=50$ using a geometrical setup as depicted in Fig. 1. We place a colloid of radius $a=5 \lambda$ in a salt solution with Debye length $\lambda =\ell _\textrm{B}$.

**Fig. 2:**
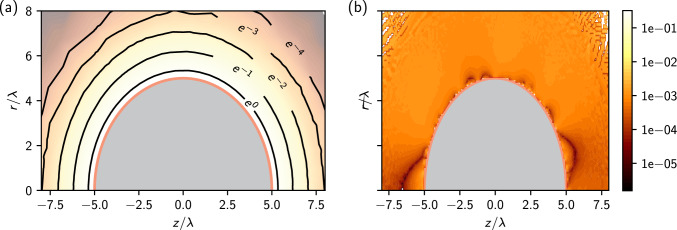
**a** IPBS solution for the potential around a single colloid of radius $R=5\lambda$, carrying a positive charge $Ze=50$ immersed in an electrolyte solution characterized by a Debye length $\lambda =\ell _\textrm{B}$. The location of the boundary involved in the iterative procedure is marked in red. **b** Relative difference between the solutions obtained with the IPBS algorithm and with standard finite elements using Neumann boundary conditions

We implemented the IPBS algorithm to solve these two-dimensional problems using the finite elements method of MATLAB, which uses piece-wise linear test functions, and in DUNE for two- and three-dimensional problems using polynomial test functions of higher degrees. We present the IPBS solution of the single colloid problem with unknown boundary conditions and the relative error with respect to constant Neumann boundary conditions in Fig. 2, showing that IPBS was able to recover the correct potential, being by all practical means indistinguishable from the solution that uses the Neumann boundary condition. The IPBS algorithm was tested using different initial configurations for the electric field at the colloid’s surface and always reached the convergence criterion $\Delta <10^{-4}$ within 5–10 iterations.

With the algorithm accuracy assessed, we can now turn to the problem of determining the potential of two like-charged colloids. We set up two colloids with charge $Ze=255$, placed at distance *x* in a medium characterized by a Bjerrum length $\ell _\textrm{B}=0.024\lambda$. The mesh setup for the finite elements calculation has the same topology as the one presented in Fig. 1. The other boundary conditions remain unchanged: the unknown electric field at the colloid surface and the Neumann condition of zero normal field on the remaining linear boundaries.

**Fig. 3:**
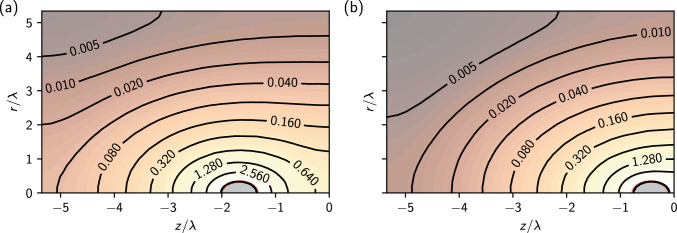
IPBS solution for the potential of the two-colloid system with radius $a=\lambda /3$ and Bjerrum length $\ell _\textrm{B} = 0.024\lambda$ for two separations, **a** $x = 10/3 \lambda$ and **b** $x=3/4\lambda$. At every isoline, the potential doubles in magnitude

Figure 3 shows some illustrative potential energy landscapes obtained with the IPBS algorithm for two different values of separation *x*, and colloids of radius $a=\lambda /3$. At large separations, where the Debye layers have, on average, a small overlap, the electrostatic influence of one colloid on the other is relatively small, and the equipotential lines around the colloid are not much perturbed. However, when the colloids are close, there is a substantial overlap of the Debye layers that perturbs the ion distribution, and the equipotential lines no longer show the original symmetry.

Within the DLVO description, the electrostatic interaction potential between two charged colloids of radius *a* separated by a distance *x* is given by:

8 $$\begin{aligned} V_{\textrm{DLVO}}(x)=Z_{\textrm{eff}}^2\ell _\textrm{B}\left( { \frac{e^{\kappa _{\textrm{eff}}a}}{1+\kappa _\textrm{eff}{}a} }\right) ^2- \frac{e^{\kappa _\textrm{eff}{}x}}{x}. \end{aligned}$$

**Fig. 4:**
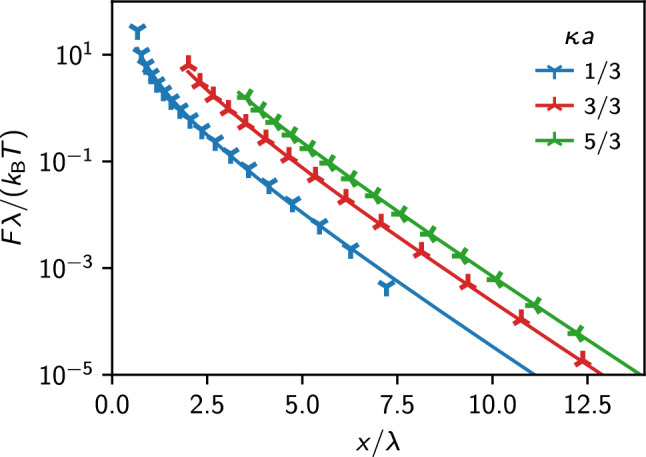
Force between two identical spherical colloids of different radii *a* (symbols) and the results of a best fit to a DLVO potential, Eq. (8), in the region $x \gg a$ (lines)

However, the validity of the DLVO description breaks down for highly charged colloidal particles due to nonlinear effects. In their seminal work [24], Alexander et al. showed, using the cell model, that it is possible to use an effective interaction of DLVO type also in the nonlinear case by considering the screening length and charge as effective parameters, $\kappa _\textrm{eff}$ and $Z_\textrm{eff}$. The basic idea is to obtain the effective parameters by solving the NBP equation for a cell model, exploiting the fact that the electric field at the cell boundary must be zero by symmetry. The effective charge density can be obtained by integration of the counter-ion cloud up to the colloidal surface [24]. Here, we will compare the effective interactions obtained from the IPBS solution with the analytical approach proposed for the fitting parameters by Aubouy et al. [25]. We note that Ref. [47] reports an approximation for the effective screening length. According to the cell model description presented in that work, we decided to perform a DLVO fit to our results only in the region $x \gg a$. Obviously, there is no rigorous way to determine which points to include in the fit. Therefore, we decided to use windows of different widths and observe the parameters’ convergence. The values of $\kappa _\textrm{eff}$ obtained from the fit always depart less than one percent from $\kappa _\textrm{eff}=\lambda ^{-1}$, in agreement with the results of Ref. [47], when the cell packing fraction tends to zero. Because of this, we decided to use only $Z_\textrm{eff}$ as a free parameter, enhancing the stability of the fit procedure. After a transient phase, the value of $Z_\textrm{eff}$ as a function of the window width oscillates around an average value, which we choose as our best estimate for the effective charge of the colloid. We present the force obtained from the fitting procedure in Fig. 4.

To investigate the validity of the analytical estimates and the cell model description, we have chosen to perform the IPBS calculation for three different values of the colloid radius, namely $a=\lambda /3$, $\lambda$, and $5\lambda /3$. These values lie outside, at the border, and within the region of validity $\kappa a \gtrsim 1$ of the analytical estimates, respectively, but in all cases, the colloid packing fraction $\eta < 5 \cdot 10^{-5}$ is well within the validity of Alexander’s prescription [26]. The force between the two colloids measured as a function of their distance is shown in Fig. 4 for the three different values of the colloid radius (squares).

The effective charge can be estimated using the analytical procedure, as [25]

9 $$\begin{aligned} Z_{\textrm{eff}} = \frac{a}{\ell _\textrm{B}}\left( 4 \kappa a t_Z + 2 \left( 5 - \frac{t_Z^4+3}{t_Z^2+1} \right) t_Z \right) , \end{aligned}$$

where the bare colloidal charge *Z* enters via

10 $$\begin{aligned} t_Z = T \left( \frac{Z \ell _\textrm{B} / a}{2 \kappa a + 2}\right) \end{aligned}$$

and

11 $$\begin{aligned} T(x) = \frac{\sqrt{1+x^2}-1}{x}. \end{aligned}$$

We report the corresponding values of $Z_\textrm{eff}$ in Table 1, together with the values obtained from the fit of Fig. 4. The DLVO potential can appropriately reproduce the force between two colloids in the far region ($x>\lambda$) and at short distances, where one could expect nonlinear effects. Moreover, the estimates for the effective charge $Z_\textrm{eff}$ are compatible with the analytical results for the single colloid in the region of validity of Alexander’s prescription.

**Table 1:** Effective charge of an isolated colloid from a fit to the DLVO potential for the numerical solution and the analytical approximation

$x/\lambda _{\textrm{D}}$	$Z_{\textrm{eff}}$ (IPBS)	$Z_{\textrm{eff}}$ (analytical)	Relative deviation (%)
5/3	$238.5 \pm 0.1$	241.4	1.1
3/3	$202.0 \pm 0.5$	208.0	2.8
1/3	$99.4 \pm 1.0$	87.2	13.9

**Fig. 5:**
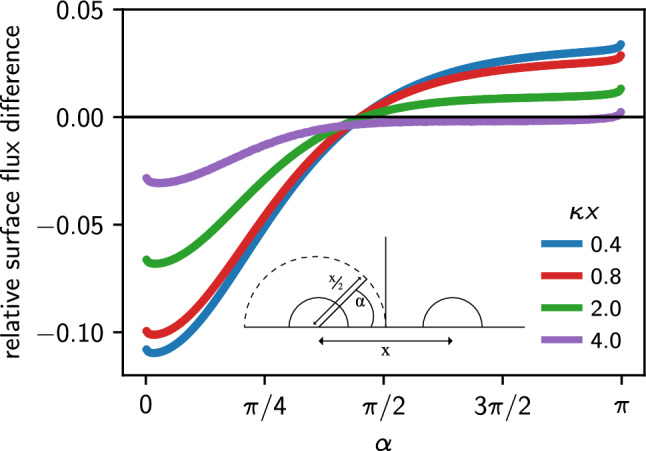
Relative deviation of the flux as a function of the angle $\alpha$ normal to the distance vector between the colloids, calculated using IPBS from the flux compared to an homogeneous normal electric field at the colloidal surface, for different distances *x* between two colloids characterized by $\kappa a = 1$ and a surface charge density $Ze/A = 0.001 e/nm^2$

To illustrate the importance of using the effective boundary conditions obtained by the IPBS algorithm, we show how these boundary conditions deviate from the assumption of a constant electric field at the surface in Fig. 5. For a homogeneously charged colloid in vacuum, the flux of the electric field through a surface element is given by $j_0 = 4 \pi \ell _\textrm{B} Ze/A$, with *A* being the surface area of the colloid. One can obtain the relative deviation by comparing this expression to the effective boundary condition obtained from Eq. (3), $(j-j_0)/j_0$. Interestingly, the distribution of the differences is not symmetric but peaked along the connecting axis. For strongly overlapping Debye layers ($\kappa x \lesssim 2$), the electric flux on the far side is increased by the presence of the second particle, whereas the two facing sides feel the mutual influence up to separations of about $\kappa x \approx 5$. For larger separations, we found that the assumption of a constant electric field all over the colloid remains valid as the value of the potential at the boundary is homogeneous within numerical accuracy.

## Including dielectric mismatch between solvent and colloids

Using unknown Neumann rather than Dirichlet boundary conditions allows us to find solutions also in the presence of a dielectric jump at the interface between colloid and solvent. Consider a space divided into two regions by a surface *S*, whose normal vectors $\varvec{{n}}$ point, by convention, outward. The internal and external regions are characterized by the dielectric constants $\epsilon _1$ and $\epsilon _2$, respectively. The boundary conditions at *S* are then

12 $$\begin{aligned} \epsilon _2\varvec{E}^{\textrm{in}} \cdot \varvec{{n}}= \epsilon _1 \varvec{E}^{\textrm{out}} \cdot \varvec{{n}}. \end{aligned}$$

Here, the field calculated in the proximity of the dielectric discontinuity is $\varvec{E}^{\textrm{in}}$ when evaluated at the internal part of *S*, and $\varvec{E}^{\textrm{out}}$ otherwise. However, implementing this boundary condition directly in a finite element method requires the solution of the Poisson problem inside the colloidal particle, which is what we avoid using our IPBS algorithm. The solution to the problem is to find an equivalent one for the NPB equation in the outer region but without any dielectric discontinuity. As an example of an equivalent problem, one can consider the same setup for the outer region but replace the inner region with one of dielectric permittivity $\epsilon _1$ (instead of $\epsilon _2$) and an induced or image surface charge density $\sigma ^{\text {ind}}$ [48]. This induced surface charge $\sigma ^{\text {ind}}$ is an unknown function of position and electric field and has to be determined for this alternative problem to be equivalent to the original one.

An implicit relation for the charge density can be derived as follows. The surface charge is related to the electric field in proximity to *S* via the Gauss law:

13 $$\begin{aligned} \left( \varvec{E}^{\textrm{out}}-\varvec{E}^{\textrm{in}}\right) \cdot \varvec{{n}} = 4\pi \sigma ^{\text {ind}}/\epsilon _1. \end{aligned}$$

We denote the charge density of the induced charges as $\sigma ^{\text {ind}}$ because a real surface charge density could also be present. In terms of finite elements *k*, we can write

14 $$\begin{aligned} \varvec{E}_k^\textrm{in}&= \varvec{E}_k^\textrm{ext} - 2\pi \sigma ^{\text {ind}}_k\varvec{{n}}_k/ \epsilon _1 \nonumber \\ \varvec{E}_k^\textrm{out}&= \varvec{E}_k^\textrm{ext} + 2\pi \sigma ^{\text {ind}}_k\varvec{{n}}_k/ \epsilon _1, \end{aligned}$$

where $\varvec{E}_k^\textrm{ext}$ is the external electric field acting on element *k*, i.e., the contribution arising from all volume and surface charges on every other element in the grid but the *k*-th one.

By combining Eqs. (12) and (13), one gets, for example,

$$ \varvec{E}^\textrm{in}_k\cdot \varvec{{n}}_k\left( \frac{\epsilon _2-\epsilon _1}{\epsilon _1}\right) = 4\pi \frac{\sigma ^{\text {ind}}_k}{\epsilon _1}. $$

By further substituting the expression for $\varvec{E}^\textrm{in}$ that appears in Eq. (14), one can directly relate the induced surface charge to the external electric field $\varvec{E}^\textrm{ext}$, as

15 $$\begin{aligned} \sigma _k = \frac{\epsilon _1}{\displaystyle 2\pi } \left( \frac{\epsilon _1-\epsilon _2}{\epsilon _{1}+\epsilon _{2}}\right) \varvec{E}^\textrm{ext}_{k}\cdot \varvec{{n}}_k . \end{aligned}$$

Since this quantity implicitly depends on the value of the induced surface charge density of all other elements through the external electric field, this equation is an implicit definition for $\sigma ^{\text {ind}}$ and can be estimated by (yet another) iterative procedure.

After estimating the surface charges, one can introduce them in the finite element scheme by noticing that they can be recast in terms of a modified boundary condition,

16 $$\begin{aligned} \frac{\partial \Phi }{\partial \varvec{n}_k}=-\varvec{E}_{k}^{\textrm{out} }\cdot \varvec{{n}}_{k}=-\left( \frac{2\varepsilon _{1}}{\varepsilon _{1}+\varepsilon _{2}}\right) \varvec{E}_{k}^{\textrm{ext}}\cdot \varvec{{n}}_{k}. \end{aligned}$$

For this purpose, we employed a second SOR procedure, starting from zero induced charge. We found that using a relatively small value for $\alpha \approx 0.2$ allows us to relax the induced surface charge density simultaneously with the unknown boundary conditions. In this way, the procedure converges within a few dozen iterations for high dielectric contrasts (like 1:80 for water–vacuum interfaces), making it a highly efficient tool for studying the influence of dielectric properties.

**Fig. 6:**
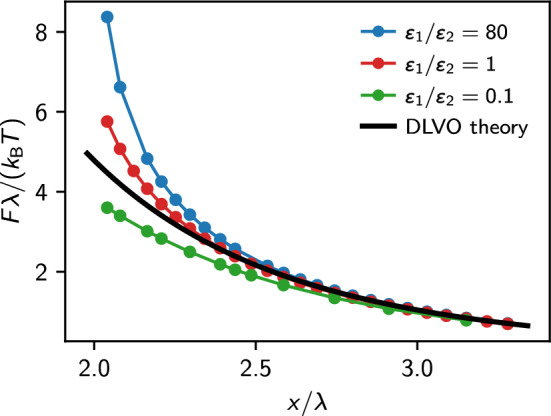
Force between two identical spherical colloids consisting of a dielectric material with (from top to bottom): low permittivity (compared to the solvent); same permittivity as the solvent; and higher permittivity than the solvent. The solid line is the DLVO force curve obtained with the analytical estimate for the renormalized charge

Figure 6 shows the obtained force–distance curves for two equivalent colloids with charge $Z=255$ elementary charges and a radius of $a=40~\text {nm}$ for three different cases, namely the situation where the colloids have the same dielectric properties as the solvent (no mismatch), for dielectric spheres with low permittivity ($\varepsilon _1 / \varepsilon _2 = 80$), which is the situation e. g. for silica particles), and for dielectric spheres with high permittivity that have a ten times higher dielectric constant than the solvent (an approximation for metallic particles in water). The salt concentration was chosen again such that $\kappa a =1$, corresponding to $60~\mu \text {mol}$ salt, and the Bjerrum length was set to be $\ell _\textrm{B} = 0.96~\text {nm}$.

The following considerations can explain the influence of dielectric contrast at the colloid’s surface. For colloids with low permittivity ($\varepsilon _2 \ll \varepsilon _1$), the induced charge density will push the electric field lines out of the colloid. According to Eq. (15), the sign of the induced charge density will be such that it counteracts the source of the external field $E_{\textrm{ext}}$ as previously defined, i.e., it tries to compensate for the field of the second particle. As a consequence, the additional force will be repulsive. Following the same reasoning for the case $\varepsilon _2 \gg \varepsilon _1$, where the electric field lines tend to be more perpendicular to the surface, and the resulting force will reduce the repulsion compared to the case without mismatch.

Figure 6 shows the resulting forces for the three distinct cases together with the DLVO prediction where the analytical estimate, Eq. (9), has been employed. The first interesting observation is that for the case without dielectric contrast, the DLVO force prediction is fulfilled precisely down to a surface–surface separation of $0.4 \lambda _{\textrm{D}}$, i.e., a region where one expects a very strong overlap of the Debye layer. This result is somewhat surprising because nonlinearities were expected to play an important role at these distances. However, it clearly shows that the concept of charge renormalization and the analytical estimate for the effective charge $Z_{\text {eff}}$ are excellent tools to model the problem of two interacting colloidal particles and that it is perfectly valid to assume $\kappa _{\text {eff}}\approx \kappa$ in our fittings above. Furthermore, the polarization contributes significantly only if the particles have a surface–surface separation of the order of one Debye length.

**Fig. 7:**
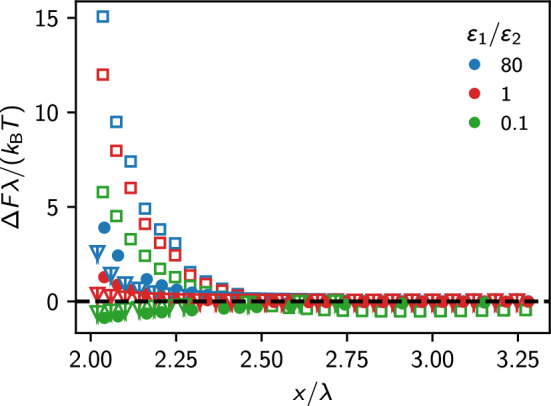
Force difference between the IPBS result and the prediction of the DLVO theory for dielectric, identical spheres of radius *a* with $\kappa a = 1/3$ (empty squares), $\kappa a = 1$ (filled squares) and $\kappa a = 5/3$ (shaded triangles). Within each set, we report three values of dielectric contrast, as in Fig. 6. The horizontal axis shows the distance measured in units of the sphere radius. We computed the DLVO potential using the analytical approximation for the charges. The dashed line marks $\Delta F=0$

**Table 2:** Salt ion number of the explicit ion MD simulations and corresponding screening parameters used for the NPB calculations

$n_s$	$\kappa$	$\kappa$ (NPB)	$\kappa$ (MD)	$Z_{\text {eff}}^\star$	$Z_{\text {eff}}$ (NPB)	$Z_{\text {eff}}$ (MD)
0	0.0333	0.011	0.010	88.3	79.8	110.0
160	0.0425	0.038	0.042	91.7	94.5	131.7
320	0.05	0.046	0.049	94.4	96.3	138.3
640	0.062	0.058	0.051	98.7	97.7	133.1
1280	0.082	0.082	0.060	105.5	105.5	126.1

Also listed are the fit parameters for the screening parameter and the effective charge after fitting a DLVO force to the results of both approaches. $Z_{\text {eff}}^\star$ denotes the predicted effective charge according to the analytical estimate. $Z_\textrm{eff}$ (MD) denotes the result from the fit to Eq. (8) reported in Fig. 9

In Fig. 7, we report the influence of the size of the colloidal particles on dielectric effects by plotting the difference between the forces calculated using IPBS and the forces predicted by DLVO theory using the analytical estimate. Again, we use $\kappa a = 1/3, 1$ and 5/3, whereas we measured the separation in units of the sphere radius. The results suggest that when the surfaces are nearly in touch, the DLVO description breaks down (this is particularly evident for the small sphere, where the net force is the largest), and at large separations, the influence of dielectric effects becomes negligible. Also, the effect of dielectric mismatches is the largest for spheres with small radii, $\kappa a \ll 1$, whereas dielectric effects vanish in the limit $\kappa a \rightarrow \infty$, where one can describe the spheres within the Derjaguin approximation as two opposing flat surfaces.

Therefore, we can expect dielectric effects to contribute significantly in solutions of charge-stabilized colloidal crystals [5] and probably even more significantly for colloids in confinement, a problem that our approach can address. In such systems, these effects might have a significant contribution and should be examined further, but this is beyond the scope of this article.

## Discussion

In Ref. [39], Kreer et al. have investigated the influence of nonlinear effects in the interactions between isolated pairs of charged colloids using explicit ion MD simulations. One of their main results was that charge renormalization seems to fail to describe the interaction between isolated pairs of colloids in the low salt limit. Here, we demonstrate that our calculations contradict these results, showing quantitative agreement with charge renormalized DLVO theory.

The MD simulation setup consisted of two colloids with charge $Z=255$ and radius $a=10~\text {nm}$ and monovalent salt ions, $Z_{ion}=\pm 1$ of radius *a*/100. Using conditions of water at room temperature ($\ell _\textrm{B} = 0.71\,\textrm{nm}$), the authors varied the number of salt ions in the explicit simulations in such a way that the total number of ions $n_\textrm{tot}$ in the simulation box was

17 $$\begin{aligned} n_\textrm{tot} = Z n_c^\prime + 2n_s, \end{aligned}$$

where $n_c^\prime$ is the number of colloids. In our finite element approach, charge neutrality is automatically fulfilled by assuming the simulation box to be in contact with a salt reservoir. This leads to a salt concentration $c_0=n_\textrm{tot} / V_\textrm{box}$ of our reservoir, whereas for the salt-free case, we chose the concentration of ions to be negligible, $\kappa \rightarrow 0$, again ensuring intrinsically the net-charge of the box to be zero. The corresponding inverse screening length is

18 $$\begin{aligned} \kappa = \sqrt{ 8 \pi l_\textrm{B}/c_0}, \end{aligned}$$

and lies between $\lambda _D = 12.5~\textrm{nm}$ and $\lambda _D = 33.33~\textrm{nm}$ (see values in Table 2).

**Fig. 8:**
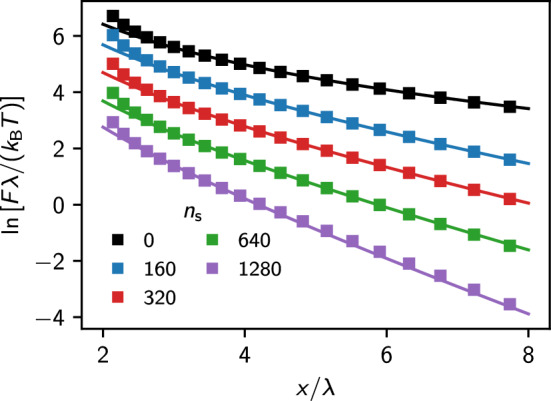
Force-distance relation (semi-logarithmic scale) of two colloids at various screening lengths obtained with IPBS (symbols) and a fit to the DLVO potential (solid lines). The values $n_s$ denote the corresponding equivalent number of explicit salt ions in the simulation box. For clarity, the values for salt concentration $n_s = 0,\,160,\,320,$ and 640 are shifted vertically by 4, 3, 2, and 1 units, respectively. Fits are performed for $x/\lambda \ge 3$

In Fig. 8, we show the logarithmic force for the different screening parameters as a function of the colloid separation. Solid lines represent the result of a fit to the DLVO potential, Eq. (8), where both $\kappa _{\text {eff}}$ and $Z_{\text {eff}}$ have been used as fitting parameters. In agreement with our previous studies, we perform the fit only in the region $x > \lambda /2$. We present the values resulting from the fit in Table 2. The solution of the NPB equation and the estimated renormalized charge show quantitative agreement, as deviations are only significant in the limit $\kappa \rightarrow 0$. We can explain these effects by the finite size of our domain. We checked this by varying the size of our domain but found the mesh we used initially to be a reasonable trade-off between computational cost and numerical stability. We observed deviations from the DLVO potential shape only at close separations, $x \lesssim a < \lambda _{\textrm{D}}$, which is expected for strongly overlapping Debye layers.

**Fig. 9:**
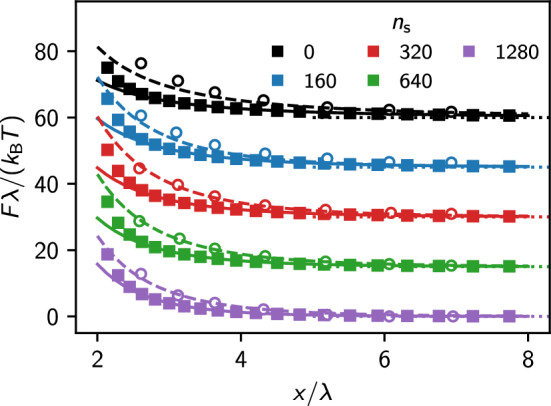
Mean force acting between the two colloids calculated with IPBS (filled symbols), MD simulations [39] (open symbols), and respective fit to the DLVO potential for the IPBS (solid lines) and MD results (dashed lines). For clarity, we shifted each data set along the vertical axis by multiples of 15 units

In their work, Kreer et al. calculated the effective charge of the colloid and found it larger than the bare one. However, such overcharging effects should not appear within the PB description. In Fig. 9, we compare the forces between the two spherical particles obtained from MD simulations data and our NPB solution. At separations $x > 2.5 \lambda$, the NPB solution agrees very well with the DLVO potential. However, also the MD simulations can be nicely described by effective DLVO interactions. Table 2 shows all our fitting parameters.

**Fig. 10:**
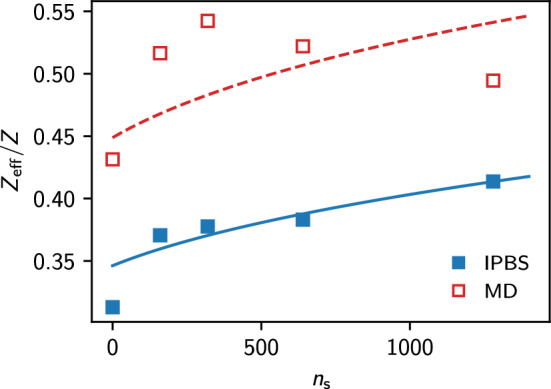
Effective charge obtained using the NPB solution and the MD simulation results. The solid line shows the prediction of the analytical estimate by Aubuoy and coworkers [47]. The dashed takes into account the effective radius of the soft-core MD simulations

Explicit ion simulations for monovalent salts for moderate charge densities have proven to agree very favorable with the results of PB theory [49, 50], so, here we set out to understand if these deviations could be artifacts of the MD simulation. In Fig. 10, we calculate the effective macro-ion charge as a function of the salt concentration, according to Fig. 3 in Ref. [39]. However, we found that in their work, Kreer et al. did not compute the effective charge $Z_\textrm{eff}$ of a DLVO interaction, but rather the factor

19 $$\begin{aligned} Z^\prime = \frac{\exp \left( \kappa a\right) }{1+\kappa a} Z_\textrm{eff} \end{aligned}$$

appearing in Eq. (8). Considering this different prefactor, the effective charges $Z_{\text {eff}}$ (MD) published in Ref. [47] agree with the values obtained from our fit and reported in Table 2 in the last column. Additionally, in Fig. 10, we show the values of the effective charge obtained that way are always less than the bare charge of the colloidal particles, confirming the validity of charge renormalization for these systems.

Still, the significant differences between the solution of the NPB equation and the results of MD simulations call for a more in-depth analysis. In particular, one should consider the presence of repulsive contributions between finite-sized macro- and micro-ions of the model interactions used in the MD simulations. In detail, Kreer et al. had chosen an exponentially decaying interaction of the form

20 $$\begin{aligned} U_{\alpha \beta } (x) = A_{\alpha \beta } \exp \left( -B_{\alpha \beta } (x-\sigma _{\alpha \beta }) / \sigma _{\alpha \beta } \right) , \end{aligned}$$

with $\alpha$ and $\beta$ denoting the different ion species and $\sigma _{\alpha \beta } = a_\alpha + a_\beta$ being the contact distance of the ions. In the NPB calculation, we can take this additional macro/micro-ion interaction also into account. Given the parameters specified in Ref. [39], $A_\textrm{CC} = 1.84~\text {eV}$ and $B_\textrm{CC} = 3.0$, this potential decays rather slowly. We found that at a separation $x=1.5a$, the interaction energy is still more than $16~k_{\textrm{B}} T$. At these energies the picture of hard spheres of radius $10~\text {nm}$, as employed by the description within a cell model surely breaks down.

Here, we map the soft-core short-ranged pair potentials onto effective hard-sphere diameters using the Barker–Henderson (BH) scheme [51],

21 $$\begin{aligned} a^\star = 1/4 \int \text {d}x\,\left[ 1-\exp (-\beta U_\textrm{CC} (x)) \right] , \end{aligned}$$

resulting in an effective colloid radius of about $13.13~\text {nm}$. We used this approximation to calculate again the effective charge, as reported in Fig. 10 using a dashed line, obtaining a greatly improved agreement with the MD simulations results. Of course, this is only a rough approximation, as it does not account for non-electrostatic effects caused by interactions between ions and between ions and colloids. In conclusion, our results support the hypothesis that this additional interaction is largely responsible for the discrepancy observed between the MD and the NPB equation results. Additionally, our data also suggests that it is meaningful to approximate the interactions in these colloidal systems with the DLVO potential using appropriate mappings for the effective radius and the analytical estimate for the effective charges.

## Conclusions

In our contribution, we have addressed the problem of the interaction between two charged colloids in an electrolyte solution using the IPBS approach, which can determine the proper boundary conditions for the NPB equation in a self-consistent way, and additionally accounting for the effect of a dielectric mismatch between colloid interior and solvent. After validating our approach with the single colloid case, we showed that the DLVO theory can correctly describe the effective interaction between isolated pairs of highly charged colloids nearly independently of the salt concentration, despite previous reports [39] claiming the opposite. Nonlinear effects show up only at distances where the Debye layers overlap significantly. In this regime, dielectric effects play an important role, leading to an additional repulsion (with respect to the case without contrast) for typical dielectric permittivity values. The dielectric repulsion is more pronounced for small colloids, vanishing in the limit of spheres with an infinitely large radius. Our IPBS method can also be used for charge-regulated boundary conditions which is left for future investigations.

## Data Availability

Data sets generated during the current study are available from the corresponding author on reasonable request.
